# Supplementary material for: HBx 128–133 Deletion Affecting HBV Mother-to-Child Transmission Weakens HBV Replication via Reducing HBx Level and CP/ENII Transcriptional Activity
Source: Viruses. 2022 Aug 26;14(9):1887. doi: 10.3390/v14091887 (PMC9506584; doi:10.3390/v14091887)
Supplement: Supplementary file 1 [file viruses-14-01887-s001.zip › viruses-1891238-supplementary.pdf]

HBx 128-133 deletion affecting HBV mother-to-child transmission weakens HBV replication via reducing HBx level and CP/ENII transcriptional activity

Yarong Song[1,2], Ying Lu[1], Yi Li[1], Minmin Liu[1], Hui Zhuang[1], Jie Li[1,*] and Jie Wang[1,2,*]

[1]Department of Microbiology & Infectious Disease Center, School of Basic Medical Sciences, Peking University Health Science Center, Beijing 100191, China.

[2]NHC Key Laboratory of Medical Immunology/Immunology Research Platform of Peking University, Beijing 100191, China.

*Correspondence: jieli@hsc.pku.edu.cn (J.L.); wangjie2015@hsc.pku.edu.cn (J.W.);

Tel.: +86-10-82805133 (J.L.); +86-10-82805137 (J.W.)

Table S1. Nucleotide sites of HBV coding regions

HBV coding region	Nucleotide sites
PreS1	nt2848-3204
PreS2	nt3205-154
S	nt155-835
PreC	nt1814-1900
C	nt1901-2452
X	nt1374-1838
RT	nt130-1161

Table S2. Sequences of primers used for amplification and sequencing of HBV X region.

Primers		Primers sequences（5’-3’）
Nested PCR for amplification	Outer primer	F: TCTGCCAAGTGTTTGCTGACGC
		R: TAGCTTGCCTGAGTGCTGTATGGTG
	Inner primer	F: CCTCTGCCGATCCATACTGCG
		R: GGTTGGAGGCTTGAACAGTAGGA
Direct sequencing		CCTCTGCCGATCCATACTGCG

F: forward, R: reverse.

Table S3. Sequences of primers used for plasmid construction

Primer names	Primers sequences（5’-3’）
Site-directed mutation F	AGGAGTTGGGGGAGGAGATTCTGGGAGGCTGTAGGCATAA
Site-directed mutation R	TTATGCCTACAGCCTCCCAGAATCTCCTCCCCCAACTCCT
pCDH-HBx-3×flag F	CGGAATTCATGGCTGCTCGGGTGTG
pCDH-HBx-3×flag R	GCTCTAGATTACTTGTCATCGTCATCCTTGTAGTCGATGTCATGATCTTTATAATCACCGTCATGGTCTTTGTAGTCGGCAGAGGTGAAAAAGTTGCAT

F: forward, R: reverse.

Table S4. Sequences of primers used for HBV DNA and HBV RNA quantification

Quantitative markers	Primers sequences（5’-3’）
HBV DNA	F: CCGTCTGTGCCTTCTCATCTG
	R: AGTCCAAGAGTCCTCTTATGTAAGACCTT
HBV 3.5 kb RNA	F: GAGTGTGGATTCGCACTCC
	R: GAGGCGAGGGAGTTCTTCT
HBV total RNA	F: AAGCCACCCAAGGCACAG
	R: GCACCAGCACCATGCAAC
ACTB mRNA	F: ACTGTGCCCATCTACGAGG
	R: CAGGCAGCTCGTAGCTCTT

F: forward, R: reverse.

Table S5. The baseline characteristics between 249 eligible mother-infant pairs and 22 randomly selected mother-infant pairs in the immunoprophylaxis success group.

		Eligible mother-infant pairs	Randomly selected mother-infant pairs	*P*
**Mothers**				
Number		249	22	
Age (years), median (range)		25.50 (18.50-37.00)	25.00 (20.00-35.00)	0.731
HBsAg (log_{10} IU/mL), median (range)		4.50 (3.40-5.22)	4.51 (3.78-4.91)	0.921
HBeAg (log_{10} S/CO), median (range)		3.15 (2.70-3.28)	3.15 (3.03-3.28)	0.641
HBV DNA (log_{10} IU/mL), median (range)		8.13 (7.01-9.11)	8.28 (7.18-8.72)	0.415
ALT (<40 U/L), n (%)		249 (100.00)	22 (100.00)	-
Genotype C2, n (%)		249 (100.00)	22 (100.00)	-
**Infants**				
Gender, male: female		130:119	9:13	0.309
Birth weight (kg), median (range)		3.40 (2.40-4.50)	3.30 (2.60-4.25)	0.295
Parturition manner, cesarean: vaginal		167:82	11:11	0.106
Feeding pattern, breast[a]: artificial		57:192	8:14	0.156
Infant's age at first dose of HepB (hours), n (%)	<12 h	237 (95.18)	22 (100.00)	0.607
	12-24 h	12 (4.82)	0	

[a]Breast-feeding included mixed feeding.

Table S6. The baseline characteristics between the immunoprophylaxis failure and success group used for analysis of HBV X region sequences.

		Immunoprophylaxis success group	Immunoprophylaxis failure group	*P*
**Mothers**				
Number		120	22	
Age (years), median (range)		24.00 (18.50-37.00)	25.50 (19.00-34.00)	0.680
HBsAg (log_{10} IU/mL), median (range)		4.49 (3.40-4.91)	4.49 (3.49-.488)	0.630
HBeAg (log_{10} S/CO), median (range)		3.15 (2.73-3.28)	3.16 (2.90-3.23)	0.946
HBV DNA (log_{10} IU/mL), median (range)		8.22 (7.15-8.96)	8.23 (7.48-8.96)	0.953
ALT (<40 U/L), n (%)		120 (100.00)	22 (100.00)	-
Genotype C2, n (%)		120 (100.00)	22 (100.00)	-
**Infants**				
Gender, male: female		60:60	13:9	0.433
Birth weight (kg), median (range)		3.40 (2.60-4.30)	3.55 (2.60-4.00)	0.212
Parturition manner, cesarean: vaginal		71:49	12:10	0.686
Feeding pattern, breast[a]: artificial		27:93	7:15	0.346
Infant's age at first dose of HepB (hours), n (%)	<12 h	112 (93.33)	19 (86.36)	0.490
	12-24 h	8 (6.67)	3 (13.64)	

[a]Breast-feeding included mixed feeding.

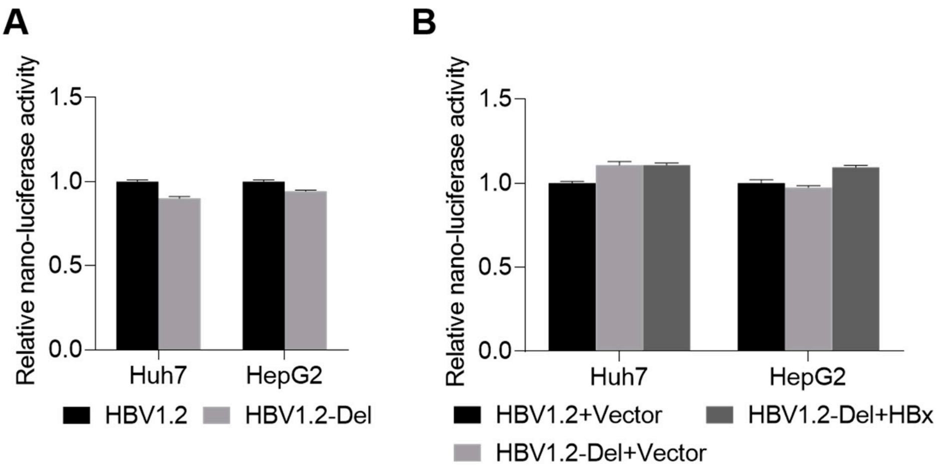

Figure S1. The transfection efficiency among different groups in Huh7 and HepG2 cells. The transfection efficiency was reflected by Nano-luciferase activity. (A) The pBB4.5-HBV1.2 or pBB4.5-HBV1.2-Del plasmid and pCDH-Nluc plasmid were co-transfected into Huh7 and HepG2 cells (5×10^5 cells/well in 6-well plate), and then the Nano-luciferase activity was detected by Nano-luciferase assays at 72 h after co-transfection. (B) The pBB4.5-HBV1.2 or pBB4.5-HBV1.2-Del, pCDH-HBx-3×flag or pCDH vector and pCDH-Nluc plasmids were co-transfected into Huh7 and HepG2 cells (5×10^5 cells/well in 6-well plate), and then the Nano-luciferase activity was detected by Nano-luciferase assays at 72 h after co-transfection.

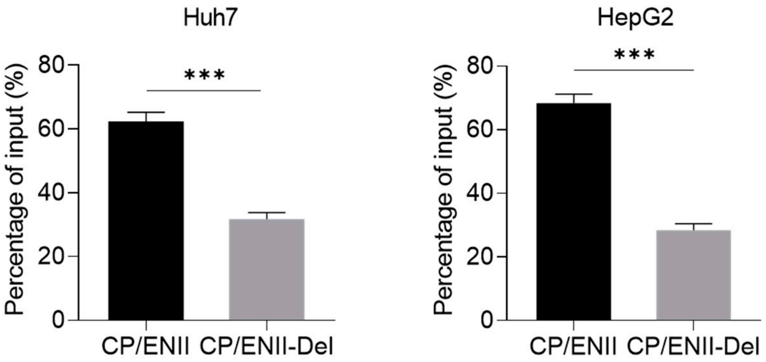

Figure S2. The effect of nt1755-1772del on HBV CP/ENII transcriptional activity. The pCDH-flag-HNF4α and pGL3-CP/ENII or pGL3-CP/ENII-Del plasmids were co-transfected into Huh7 and HepG2 cells (3×10^6 cells/well in 10 cm dish), and then the binding capacity between HNF4α and HBV CP/ENII was detected by ChIP-PCR at 48 h after co-transfection. The relative amount of chromatin enrichment (percentage of the input) in ChIP-PCR was analyzed by gray value analyses. ***P<0.001, Student's *t*-test.
